# Effects of Aletris Farinosa

**Published:** 1861-01

**Authors:** J. P. Dromgoole

**Affiliations:** Chulahoma, Mississippi


### ART. IV.—*Effects of Aletris Farinosa.* By J. P. DROMGOOLE, M.D., of Chulahoma, Mississippi.

ALETRIS FARINOSA, also known by the popular names of helonia dioicia, false unicorn-root, star-wort, blazing star, colic-root, star-grass, unicorn-root, and star-root, is a small plant, found in shady and hilly situations in most parts of the United States. It is more generally designated as "unicorn" or "star-root," and for a considerable time it has been held in high repute by many of the officious and communicative old women of the country. Notwithstanding the great variety of names by which it is known would seem to attach considerable importance to its medicinal properties, yet its therapeutical effects are apparently unknown to the medical profession. I have used it very extensively during the past eight years, and its administration has been followed by the most happy effects, in many cases proving itself superior to other remedies. Dr. Bigelow states that he knows of no plant which surpasses it in genuine, intense, and permanent bitterness. From that opinion I partially dissent, as I find it a mild, permanent, and rather pleasant bitter, but not so intense as colombo, quassia, or gentian.

The root, which is the part used, is small and bulbous, extremely hard and flinty, and in its action it is emmenagogue, tonic, alterant, and slightly stimulating. I have found very few physicians who have ever prescribed a dose of it—many never having heard of it.

Considering its valuable remedial effects, and a general ignorance in regard to any medical properties it may contain, I have been induced to call attention to so potent a remedy, hoping others more prominently situated may give it a fair trial. I am confident it deserves a trial, and think it should hold an important place in our pharmacopœia. I have used it successfully in the treatment of amenorrhœa, dysmenorrhœa, leucorrhœa, hysteria, chlorosis, and other diseases connected with the organs of gestation, and have found it to be the most effectual remedy I have as yet employed.

I am not prepared to say that it is a specific or direct emmenagogue, yet its administration has produced results which follow the use of no other medicine. For instance: cases of amenorrhœa of several months or years standing, which have baffled the skill of eminent physicians, and failed to yield to all known remedies, have proved perfectly tractable upon a free use of star-root. For all female irregularities, I find it to be unsurpassed in its certain and efficient effects.

All female or uterine disorders, attended with a feeble atonic condition of the system, particularly call for this remedy. Of course, the remedy will fail in cases of amenorrhœa attended with uterine engorgement, or some accidental or congenital fault of the organ of gestation.

The same may be said of the other diseases above mentioned. A few doses will do no good; it must be used regularly and perseveringly, and it will gradually produce a change in those structures from which the menstruæ flow.

In my hands it has proved more valuable in female complaints than all other remedies combined, and I hope its effects may cause many physicians to hail its introduction with a degree of satisfaction which no emmenagogue has ever yet enjoyed. I generally employ the following combination as the prevailing symptoms indicate its use:—

℞.—Aletridis Farinosæ, ℥xvj;
Fol. Anthemid. nob.
" Buchu,
Rad. Sarsap. āā ℥ij;
Aquæ Bullient.
Best Whisky, āā Ovj.

Bruise all the ingredients well, mix thoroughly, and place in a percolator. Pour on one-half of the water and whisky; let the mixture stand twelve hours, displace, and set the solution aside. Displace the remaining half several times while hot. Mix the two solutions, and by means of a water or sand bath evaporate to one gallon. Dose, one tablespoonful after each meal. It may also be given in powder, in doses of from ℈j to ʒj after each meal.

In feeble constitutions, and where the stomach is considerably deranged, I frequently employ the following compound tincture:—

℞.—Aletridis Farinosæ, ℥iij;

Fol. Buchu, ℥ss;

Rad. Serpentariæ, ℥ij;

Sem. Cardam.

Cort. Aurant. āā ʒj;

Spts. Rectif. dil. Oij.

Bruise, mix, and digest for fourteen days, and filter; or displace. Dose, one tablespoonful half an hour after each meal. If the patient be pregnant, or if there be considerable uterine engorgement, I consider the star-root contra-indicated.

I have never known abortion produced by it, yet such complaints from patients have been made as to induce me to withhold the medicine in many instances.

In order to be certain of procuring a good article, buy the root before being pulverized, as I have often been disappointed in employing it as ordinarily found in drug stores.

In chlorosis I use carbonate of iron in connection with the above, paying strict attention, in all instances, to the secretions, diet, exercise, state of the mind, external applications, and sympathetic affections. At present I am not prepared to say anything in regard to its *modus operandi*, hoping others may, after a fair trial, be induced to point out its more direct physiological effects upon the system.

